# When the value of gold is zero

**DOI:** 10.1186/1756-0500-7-404

**Published:** 2014-06-27

**Authors:** J Geoffrey Chase, Knut Moeller, Geoffrey M Shaw, Christoph Schranz, Yeong Shiong Chiew, Thomas Desaive

**Affiliations:** 1 Center for Bio-Engineering, University of Canterbury, Private Bag, 4800 Christchurch, New Zealand; 2 Institute of Technical Medicine (ITeM), Furtwangen University, Villingen-Schwenningen, Germany; 3 Department of Intensive Care, Christchurch Hospital, Christchurch, New Zealand and Department of Medicine, University of Otago, Christchurch, New Zealand; 4 GIGA-Cardiovascular Sciences, University of Liege, 4000 Liege, Belgium

**Keywords:** Gold standard, Mechanical ventilation, Research methodology, Validation, Clinical trials, Animal trials

## Abstract

This manuscript presents the concerns around the increasingly common problem of not having readily available or useful “gold standard” measurements. This issue is particularly important in critical care where many measurements used in decision making are surrogates of what we would truly wish to use. However, the question is broad, important and applicable in many other areas.

In particular, a gold standard measurement often exists, but is not clinically (or ethically in some cases) feasible. The question is how does one even begin to develop new measurements or surrogates if one has no gold standard to compare with?

We raise this issue concisely with a specific example from mechanical ventilation, a core bread and butter therapy in critical care that is also a leading cause of length of stay and cost of care. Our proposed solution centers around a hierarchical validation approach that we believe would ameliorate ethics issues around radiation exposure that make current gold standard measures clinically infeasible, and thus provide a pathway to create a (new) gold standard.

## The gold standard

Medicine is dominated by measurements. Behind virtually every decision lurks one or more measurements of one or more physiological or biochemical parameters. Although it is sometimes less directly acknowledged, there is the obvious significant impact of the quality of the measurement on the ability to deliver the desired quality of care.

Thus, for most measurements in medicine, there exists one or more “gold standards”. While there is debate over the definition [1], “gold standard” here simply represents the best available measurement of a parameter, even if it is not the most cost effective or clinically feasible. These gold standards are the metrics to which any new measurement is compared, as well as the means by which potential variability in study outcomes can be assessed.

Incorporating evidence-based medicine into current clinical practice is a main avenue for optimizing care [2]. However, this goal has proven very difficult in critical care medicine, where there are numerous examples of clinical trials yielding conflicting results or failing to deliver clear results. It bears repeating that measurements are at the heart of the intervention and outcome assessment in these clinical trials. The practice of critical care, perhaps more than any other specialty, is driven by metrics that are surrogates of pathophysiological processes. Diagnostics in critical care are not primarily laboratory based, as they are in oncology or hematology for example. Thus, one can test for cell markers in types of lymphoma, but there are no gold standard diagnostics to quantify pathophysiological processes seen in acute respiratory distress syndrome (ARDS) and septic shock. Yet, managing these conditions and other pathophysiological derangements are the ‘bread and butter’ of critical care.

Mechanical ventilation (MV) in ARDS is one area for which there is no well-accepted approach to care, in particular, positive end expiratory pressure (PEEP) selection, with several conflicting randomised trial results [3]. The main goals of MV are to support patient breathing and, where possible, select a PEEP that safely maximises recruitment. Thus, while clinical outcome variables, such as mortality or length of MV, can be linked to a given approach or method, there is no guarantee that the intervention being tested achieved the recruitment desired [4].

Fortunately, there is a relatively well-accepted gold standard measurement of recruitment, the Computed Tomography or CT scan, that is currently the most accepted, if not fully proven, metric for titrating care [5]. Certainly, one or more CT slices of the lung can clearly show the level and amount of recruitment [6], providing clear, direct and effective measurement of the immediate clinical outcome and impact of an intervention. It has been used in animal studies [7,8] and some human studies [6] to assess recruitment, although it is not effective for repeated dynamic assessments.

In particular, there is increasing opposition to using, or overusing, radiation medical imaging, and CT in particular, even in research studies [9,10]. Equally, CT has never been very practical, nor cost effective, in clinical practice for assessing recruitment. Just moving the patient and having to ventilate using a longer breathing circuit can significantly impact pulmonary mechanics and patient condition. Finally, it is not only impractical to use CT to make frequent adjustments of PEEP (up to 4 times daily), but it would also expose patients to unacceptably high doses of radiation.

Thus, the field is left without any gold standard that can effectively or ethically be used in care or research. This latter use is critical, because without a high quality gold standard that is also clinically feasible, it is not possible to safely or effectively translate animal studies or similar results into human studies and improved outcomes. As a result, emerging non-invasive approaches, such as electrical impedance tomography (EIT) [11] and model-based methods [12], cannot even be assessed to determine their efficacy in humans. This failure leaves these modalities stranded without a means of validation in human subjects. More specifically, when there is no gold standard direct measurement, there is no way to prove its replacement or surrogate has equal or effective clinical value.

### A path forward

**Figure 1:**
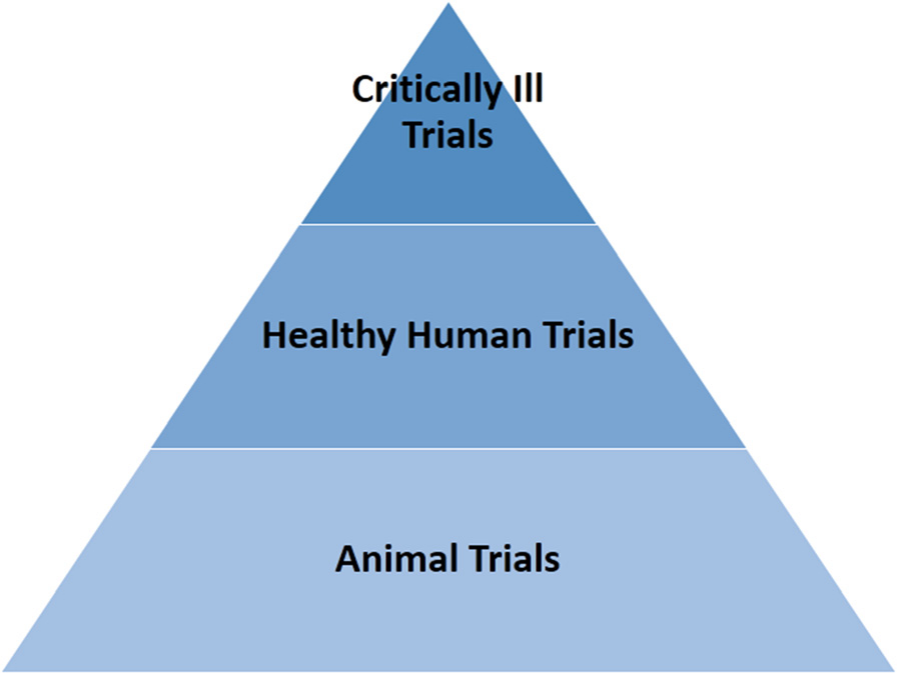
**A simple 3-phase validation pathway.**

What would be useful is a validation roadmap by which new methods could be assessed safely and ethically. Figure 1 shows a 3-phase validation approach with the narrowing pyramid indicating that potential for fewer methods to pass each stage. The end goal is validation on critically ill cohorts, but only subsequent to proof that any new method can:

• Accurately capture recruitment and recruited volume in the heterogeneous ARDS lung.

• Work effectively in humans.The specific phases in Figure 1 are rationalized:

1. Animal Trials: allow a heterogeneous ARDS lung to be induced and for a method to be tested over the evolution of the disease state. Several CT slices can be taken at each step without concern over radiation dose. Several slices enable any method to accurately quantify error and error relative to the variability across the CT slices.

2. Healthy Human Trials: allow any method to show it works in humans. Given recent evidence that modest recruitment can be obtained in the healthy human lung with small added pressure, this validation should be enough to show ability to work with human anatomy. In these trials, only a single CT slice might be taken to minimise radiation. Healthy humans are chosen for this level because they can tolerate a small radiation dose with the minimum possible risk.

3. Critically Ill Trials: the final validation on MV patients, using only a single CT slice as required as final validation of any concept that passes the first two phases.

Thus, using this approach, there are no trials on critically ill patients and no significant added risk until a new method is proven in animals (no added human risk) and healthy humans (no significant added human risk).

### Some final thoughts

So, where does this situation leave us? Perhaps the gold standard does not exist. Perhaps, we should abandon the ‘gold standard’, as governments did in the 1930’s, for a more adaptive method that can provide real-time metrics that could be ultimately compared in large well designed randomised trials. Or possibly, we should create a consensus or agreed pathway to creating a true gold standard to enable better randomised trials and thus better care.

Otherwise, these issues leave the field without measurements that are critical for providing and, especially, improving care. It is a general problem, what do you do when the value of (your) gold (standard) is zero?

### Ethics committee approval

Not applicable, none was required for this comment.

## Abbreviations

ARDS: Acute respiratory distress syndrome; CT: Computed tomography; EIT: Electrical impedance tomography; MV: Mechanical ventilation.

## Competing interest

The authors declared that they have no competing interest.

## Authors’ contributions

All authors contributed to the intellectual content and writing of this comment. All authors read and approved the final manuscript.
